# Restriction site free cloning (RSFC) plasmid family for seamless, sequence independent cloning in *Pichia pastoris*

**DOI:** 10.1186/s12934-015-0293-6

**Published:** 2015-07-14

**Authors:** Thomas Vogl, Mudassar Ahmad, Florian W Krainer, Helmut Schwab, Anton Glieder

**Affiliations:** Institute of Molecular Biotechnology, Graz University of Technology, Petersgasse 14, 8010 Graz, Austria; Queensland University of Technology, 2 George St., Brisbane, QLD 4000 Australia

**Keywords:** Protein tagging, Protein tags, Seamless cloning, *Pichia pastoris*, Expression optimization, Cloning strategy, Type IIS restriction endonucleases

## Abstract

**Background:**

Tagging proteins is a standard method facilitating protein detection, purification or targeting. When tagging a certain protein of interest, it is challenging to predict which tag will give optimal results and will not interfere with protein folding, activity or yields. Ideally, multiple tags and positions are tested which however complicates molecular cloning and expression vector generation. In conventional cloning, tags are either added on PCR primers (requiring a distinct primer and PCR product per tag) or provided on the vector (typically leaving a restriction site scar).

**Results:**

Here we report a vector family of 40 plasmids allowing simple, seamless fusions of a single PCR product with various N- and C-terminal tags, signal sequences and promoters. The restriction site free cloning (RSFC) strategy presented in this paper relies on seamless cloning using type IIS restriction endonucleases. After cutting out a stuffer (placeholder) fragment from the vectors, a single PCR product can be directly inserted in frame into all 40 plasmids using blunt end or TA ligations, requiring only verification of the orientation. We have established a RSFC vector family for the commonly used protein expression host *Pichia pastoris* and demonstrated the system with the secretory expression of horseradish peroxidase (HRP). HRP fusions to four tags (Myc, FLAG, His, Strep) and two fusion proteins (GFP and MBP) showed a 31-fold difference in volumetric activities. C-terminal tagging caused in some cases almost a complete loss of function, whereas N-terminal tags showed moderate differences.

**Conclusions:**

The RSFC vectors provide an unprecedented toolbox for expression optimization in *P. pastoris.* The results obtained with HRP underline the importance of comparing different tags to maximize activities of fusion proteins. In a similar fashion the RSFC strategy can be applied in other expression hosts to screen for optimal promoters, signal sequences or to facilitate the evaluation of (iso-) enzyme families.

**Electronic supplementary material:**

The online version of this article (doi:10.1186/s12934-015-0293-6) contains supplementary material, which is available to authorized users.

## Background

Protein tags are commonly applied tools facilitating purification (affinity tags), enabling immuno-detection (epitope tags) or increasing solubility. Fusions to fluorescent proteins help elucidating the cellular localization and fusions to signal sequences provide specific intracellular targeting or secretion [1, 2]. However, as an extrinsic addition to a protein of interest (POI), such fusions may also show detrimental effects by affecting protein conformation, yields, activity or stability [1, 3]. The specific interactions of the POI with a certain tag are generally hard to foresee and may also depend on the position of the tag (N- or C-terminal). Unknown proteolytic processing or intracellular targeting of the POI may also influence the suitability of a specific fusion site. In addition, the same tagged protein may behave differently depending on the host system used (e.g. bacteria, yeast, higher eukaryotes) [4]. As there are large numbers of affinity, epitope tags and fusion proteins available it is challenging to predict the optimal choice for a certain POI. Therefore, commonly multiple tags are tested in N- or C- terminal positions and screened for optimal results [4–7].

However, preparing expression constructs containing multiple tags may require tedious cloning work. Tags are commonly provided on the plasmid adjacent to the multiple cloning site (MCS). This requires unique vectors for each tag and N-/C-terminal position. The gene of interest (GOI) needs to be cloned into the MCS via unique restriction endonuclease (RE) recognition sites. These restriction site scars remain in the protein coding sequence (CDS) and are later translated into additional amino acids, which may interfere with the POI’s properties. Also cloning strategies based on recombination such as Gateway (e.g. [8]) leave the recombination sequence as a scar in the CDS.

Ideally, tags should be fused seamlessly to the GOI i.e. without any restriction site scars or additional sequences from the MCS. Seamless cloning can be achieved by various strategies [9]. Frequently, tags are directly added by PCR as a 5′ overhang of a primer and thereby seamlessly attached to the CDS. This approach requires however a unique primer for each tag, N-/C-terminal position and each GOI.

We aimed to design a simple, seamless system to facilitate testing of multiple tags in N-/C-terminal position at minimal cost and effort (e.g. without the need to order numerous primers).

Several novel cloning methods are completely independent of REs and allow simple assembly of multiple fragments solely by short overlaps (around 25 bp) relying on in vitro ‘recombination’ (e.g. annealing of single stranded overhangs and enzymatic linkage). These methods include SLIC (sequence and ligation–independent cloning) [10], SLiCE (Seamless Ligation Cloning Extract) [11], Gibson assembly [12], CPEC (Circular Polymerase Extension Cloning) [13] and are concisely compared on the website of the Joint BioEnergy Institute (JBEI), Emeryville, CA, USA [14, 15]. All these methods may be used to seamlessly add a tag to a protein by adding the tag sequence to a PCR primer. However, there is an additional overhang required for in vitro recombination with the vector, requiring relatively long primers. Most inconveniently a new primer is needed for each tag, each position and each POI to be tested.

**Figure 1:**
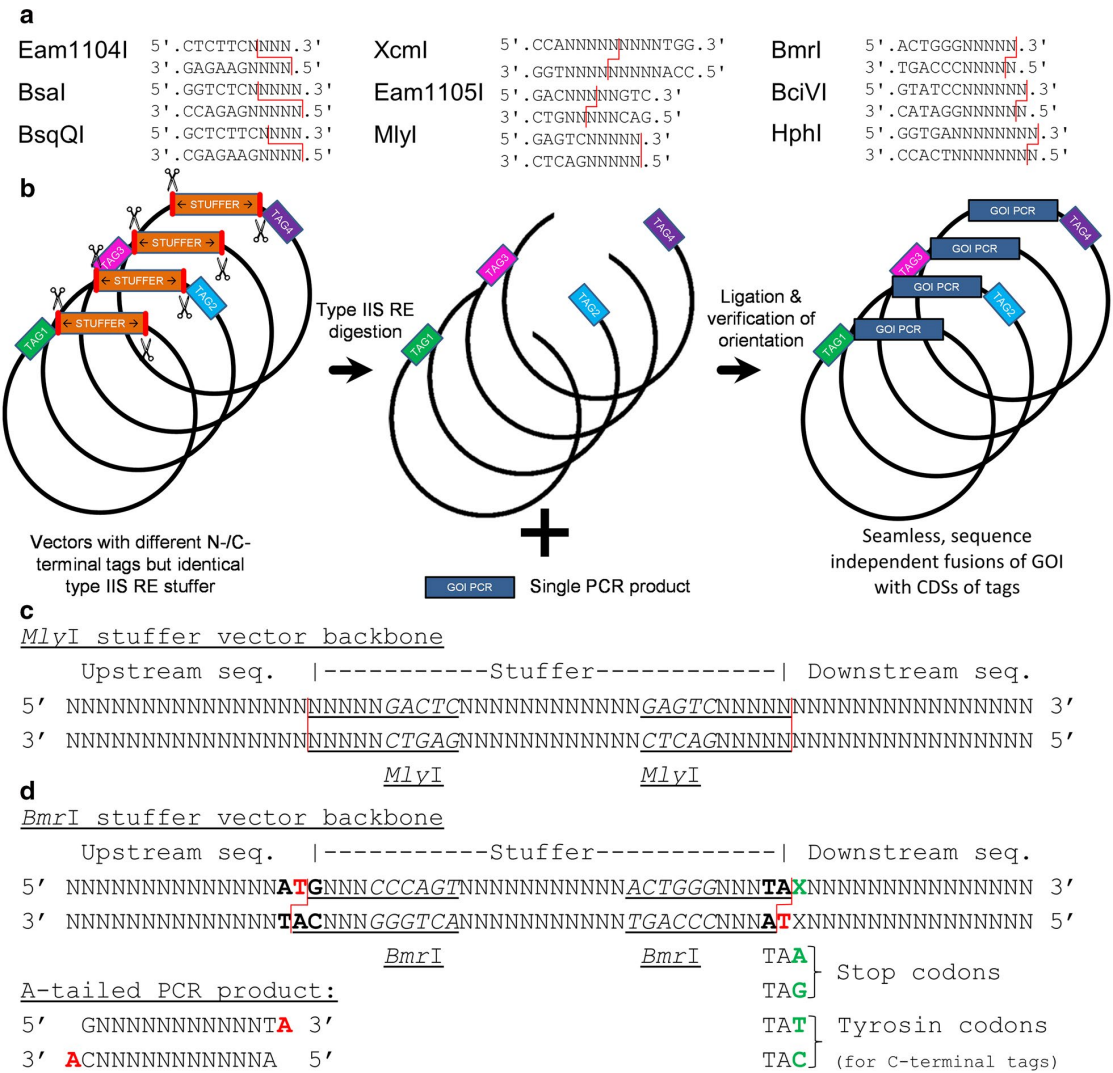
Detailed outline of the restriction site free cloning (RSFC) strategy. **a** Recognition sites of various type IIS REs. The cleavage patterns are indicated as *red lines*. **b** Schematic workflow of restriction site free cloning. After removal of a stuffer fragment using type IIS REs, a single PCR product can be ligated into all vectors in a seamless, sequence independent fashion. The strategy is shown for four vectors but can be extended to as many as desired. **c** Design of the *Mly*I stuffer fragment for blunt end ligations. The *Mly*I recognition sequence is written in italics, the entire cleavage pattern is underlined. Variable bp are denoted as ‘N’. *Upstream* sequences may include promoters, N-terminal tags and signal sequences, downstream sequences may include C-terminal tags and stop codons. **d** Design of the *Bmr*I stuffer fragment for TA cloning. Same explanation as (**c**), in addition the incorporation of the dA and dT residues for TA cloning via Start- and Stop/Tyr-codons are shown (*red*). By varying the last nucleotide ‘X’ of the Stop/Tyr codon, either translation can be terminated or a C-terminal tag linked in frame. A dA-tailed PCR product suitable for ligation is also shown.

Therefore we have based our strategy on type IIS REs. In contrast to type II REs, which recognize and cut within a palindromic sequence, type IIS REs cut outside of a non-palindromic recognition sequence [16, 17]. Thereby RE site scars can be circumvented making type IIS REs prominent tools for seamless cloning [9]. There are various type IIS enzymes available that create different types of overhangs including up to 4 bp overhangs suitable for sticky end cloning (e.g. *Eam*1104I [18], *Bsa*I [19, 20]), single base pair overhangs that can be applied for TA cloning (e.g. *Xcm*I [21, 22], *Eam*1105I [23, 24], *BciVI* [25]) or blunt end cloning (*Mly*I/*Sch*I [25, 26]), see Figure 1a.

In this study we have evaluated type IIS REs for blunt end and TA cloning and designed a restriction site free cloning (RSFC) strategy that enables simple, seamless cloning of a PCR product in frame with any desired upstream or downstream sequence in a vector. Based on this strategy, we have designed a RSFC vector family of 40 plasmids for the methylotrophic yeast *Pichia pastoris*, a commonly used protein production host for industrially relevant biocatalysts and biopharmaceuticals [27–29]. The vectors feature different epitope and affinity tags (Myc, FLAG, His, Strep) and fusion proteins (eGFP and MBP) in N- and C-terminal position that are provided for intracellular and secretory expression.

## Results and discussion

### Restriction site free cloning (RSFC)

#### Blunt end vs. TA cloning concept

We aimed to design a vector system in which a single PCR product of a GOI can be directly fused, sequence independently to various N- or C-terminal tags provided on different plasmids. Thereby only two primers are required to test seamless fusions of multiple tags with the GOI. This design is achieved by inserting a stuffer (placeholder) fragment flanked by two type IIS RE sites in opposite orientations in all vectors (Figure 1b–d). The CDSs of different N- and/or C-terminal tags or fusion proteins are provided upstream/downstream of the stuffer fragment. By digestion using the respective type IIS RE, the stuffer fragment including the RE sites is cut out, resulting in RE site free vector backbones that can be directly ligated with the same PCR product (Figure 1b).

Commonly used type IIS RE based cloning strategies such as Golden Gate cloning [19, 20] cannot be used for this purpose as they rely on type IIS enzymes creating short overhangs such as *Eam*1104I or *Bsa*I (Figure 1a). The use of these enzymes requires also RE digestion of the PCR product and the overhangs created on the vectors would differ between tags and impede seamless fusions.

Direct, sequence independent cloning of PCR products is in this context only possible by using TA cloning or blunt end ligations. These methods are in general not directional (with a few exceptions e.g. [25, 26]) and require verification of the orientation (e.g. by colony PCR, cPCR). TA cloning is based on the property of *Taq*-Polymerase to add a single deoxyadenine (dA) nucleotide at the 3′ ends of amplified DNA [21]. These PCR products can be directly cloned using a vector with a single 3′ deoxythymidine (dT) overhang. TA cloning works more efficiently than blunt end cloning [21], however the required dA nucleotide complicates seamless fusions to tags as it must be universally incorporated in the transitions between tag and vector. In this respect, blunt end ligations, that are completely sequence independent, are more favorable.

We designed test vectors based on type IIS REs for blunt end and TA cloning to compare their suitability. There is only one blunt end type IIS RE available that cuts outside of its recognition sequence (*Mly*I). *Mly*I has also been established for directional blunt end ligations of PCR products using a *lac*O, *lac*Z based blue-white screening in *Escherichia coli* [25, 26]. There are several type IIS REs available, that create a single base 3′ overhang (e.g. *Bmr*I, *Bci*VI, *Hph*I, see Figure 1a). We tested commercially available preparations of these three enzymes all of which showed sufficient cleavage efficiencies (data not shown). *Hph*I and *Bci*VI have been previously used for TA cloning [21, 25], yet these restriction sites were present more frequently in the vector backbones we wanted to use. Therefore we used *Bmr*I.

The basic sequence design of the transitions between the vector, the type IIS restriction sites and the stuffer fragment are shown in Figure 1c, d. For blunt end cloning using *Mly*I, the design is completely sequence independent (Figure 1c). For TA cloning, 3′ dT residues must be provided on the vector backbone and incorporated in the transition between vector and GOI. We solved this by using the dT nucleotide of the start codon (ATG) and the dA nucleotide of a partial stop codon (TAX), creating a 3′ dT base on the reverse strand (Figure 1d). Depending on the desired sequence context, ‘X’ may be provided on the vector side as A/G for a stop codon (translation termination) or T/C (coding for tyrosine, for linkage of C-terminal tags).

#### Cloning efficiencies

We compared the basic blunt end and TA cloning based system at first with expression vectors for *Schizosaccharomyces pombe* as these plasmids required fewer modifications in the vector backbones than the *P. pastoris* plasmids we intended to use. See Additional file 1: Figure S1 for plasmid maps and the “Materials and methods” section for details on the design. After cutting out the stuffer fragment using *Mly*I or *Bmr*I, the vector backbones were dephosphorylated to counter act self-ligation. Primers for insert amplification were phosphorylated prior to ligation (see “Materials and methods” section for experimental details and a simple, cost effective protocol). Both cloning strategies resulted in similar transformation efficiencies (via electroporation), approximately 10^2^–10^3^ colony forming units (cfu)/µg DNA (in the ligation reaction) with self-made competent cells (competence with circular, supercoiled plasmids: 10^6^–10^7^ cfu/µg DNA) and in both cases all 10 out of 10 clones tested contained an insert. We verified the orientation by cPCR; as statistically expected approximately half the clones contained an insert in the correct orientation (blunt end/*Mly*I: 5 of 10, TA cloning/*Bmr*I: 7 of 10). Additional file 2: Figure S2 outlines a simple cPCR strategy to test the correct orientation (using sequencing primers of the vector and the primers used for amplifying the insert). The vector/insert transitions were confirmed by sequencing and did not show any mutations. However, when cloning an insert into a larger set of vectors using blunt end ligations (see *P. pastoris* vectors below) we noticed occasionally single bp deletions of the insert adjacent to the vector transition (<5% of constructs). Sequencing of additional transformants resulted in all cases in correct sequences. Notably, the deletions were always in the 5′ ends of the insert and occurred more often after repeated freeze/thaw cycles of the PCR product. We therefore recommend aliquoting the PCR product and vector backbones and using them only once.

In general these RSFC ligations resulted in lower efficiencies (cfu/µg DNA) than comparable sticky end ligations, but still yielded sufficient numbers of transformants for our standard cloning applications. *Mly*I based blunt end ligations worked similarly efficient as *Bmr*I based TA cloning. Previously, TA cloning has been reported to be more efficient than blunt end cloning [21], however the difference may arise from the different enzymes used for vector preparation in our study.

We mutated *Mly*I sites present in the vector backbones to enable the stuffer removal (see “Materials and methods” section for details). All mutations but one resulted in no differences in DNA yields compared to the parental plasmids. Mutating a *Mly*I site in the *E. coli* pUC origin of replication to a sequence previously reported [25, 26] decreased plasmid yields to approximately 30% of the unmutated parental plasmid (wildtype pUC: ~400 ng/µl, *Mly*I mutated pUC: ~120 ng/µl). The *MlyI* site appears also in other high copy number origins of replication (ori) and switching to a lower copy number *ori* would also result in lower plasmid yields. We intended to use the RSFC plasmids only for sub cloning and aimed to perform expression in *P. pastoris*. To this end mini prep yields (typically >5 µg) were sufficient. However if similar plasmids should be used for expression in *E. coli*, we would recommend to screen other possible mutations of the *Mly*I site using degenerate primers to restore wild type plasmid yields.

However, the blunt end/*Mly*I based strategy allowed completely seamless cloning whereas seamless TA cloning was hindered by the requirement for dT/dA bases in the insert/vector transition. This problem is similar to the use of typeIIS enzymes creating longer sticky end overhangs that need to be complementary between all plasmids of a vector family (for example in plasmids by BioGrammatics, Inc., Carlsbad, CA, USA and ‘Electra’ plasmids by DNA2.0, Inc., Menlo Park, CA, USA). As outlined in Figure 1d, the TA strategy can be still used for fusion of the same PCR product to different tags, however N-terminal tags are always linked via an ATG (coding for methionine/start), whereas C-terminal tags must be linked via tyrosine codons. Tyrosine is naturally a relatively rarely occurring and bulky amino acid, making it structurally rather unfavorable as a linker to an adjacent tag. In ‘Electra’ plasmids by DNA2.0 this issue is solved by adding an additional C-terminal ‘linker’ amino acid to all vectors, whereas in the RSFC strategy only vectors with C-terminal tags require a linker amino acid. Still we have solely focused on the blunt end/*Mly*I based strategy in the following plasmid design for *P. pastoris*. The blunt end/*Mly*I based ligations required no A-tailing step of PCR products but reached similar ligation efficiencies as TA cloning and allowed completely sequence independent fusions.

In summary, our cloning approach, relying on blunt end or TA ligations between a phosphorylated PCR product and a dephosphorylated vector backbone created by type IIS RE digestion, allowed seamless, sequence independent cloning at reasonable efficiencies. PCR products can be directly used for ligations and do not need RE digestion, therefore any insert sequence can be used (TA cloning with proof reading polymerases requires a separate dA-tailing step). There have previously been type IIS based cloning efforts using blunt end and TA ligations for the cloning of PCR fragments [21–26]. However, these strategies did not allow seamless fusions and are in part with *lac*O, *lac*Z based blue white screening [25, 26], despite the convenience of directional cloning, even incompatible with seamless fusions. To distinguish our approach from these efforts and other type IIS based strategies such as Golden Gate cloning [19, 20], we have termed our approach restriction site free cloning (RSFC).

### RSFC plasmids for *P. pastoris* as toolbox for optimizing protein production

#### Tags and fusion proteins

We applied the RSFC cloning strategy to design a plasmid family for *P. pastoris* allowing seamless fusions of a GOI with various tags and fusion proteins in N- and C-terminal position. There are different expression plasmids available for *P. pastoris* based on various cloning strategies such as Gateway [8], TA cloning [22, 25], sticky end type IIS ligations (plasmids by BioGrammatics, ‘Electra’ plasmids by DNA2.0) and ‘classical’ typeII RE/ligation based systems ([30–32] and *P. pastoris* plasmids by Life Technologies, Carlsbad, CA, USA). The pCri vector family [32] is a multi-host platform, allowing to clone a single PCR product via restriction digestion and a MCS into different vectors. For *P. pastoris* only three pCri plasmids with a His tag are available. Therefore none of the vector systems currently available for *P. pastoris* offer different tags and only the BioGrammatics and Electra plasmids by DNA2.0 vectors allow seamless, yet sequence dependent cloning still requiring restriction digestion of the insert.

**Figure 2:**
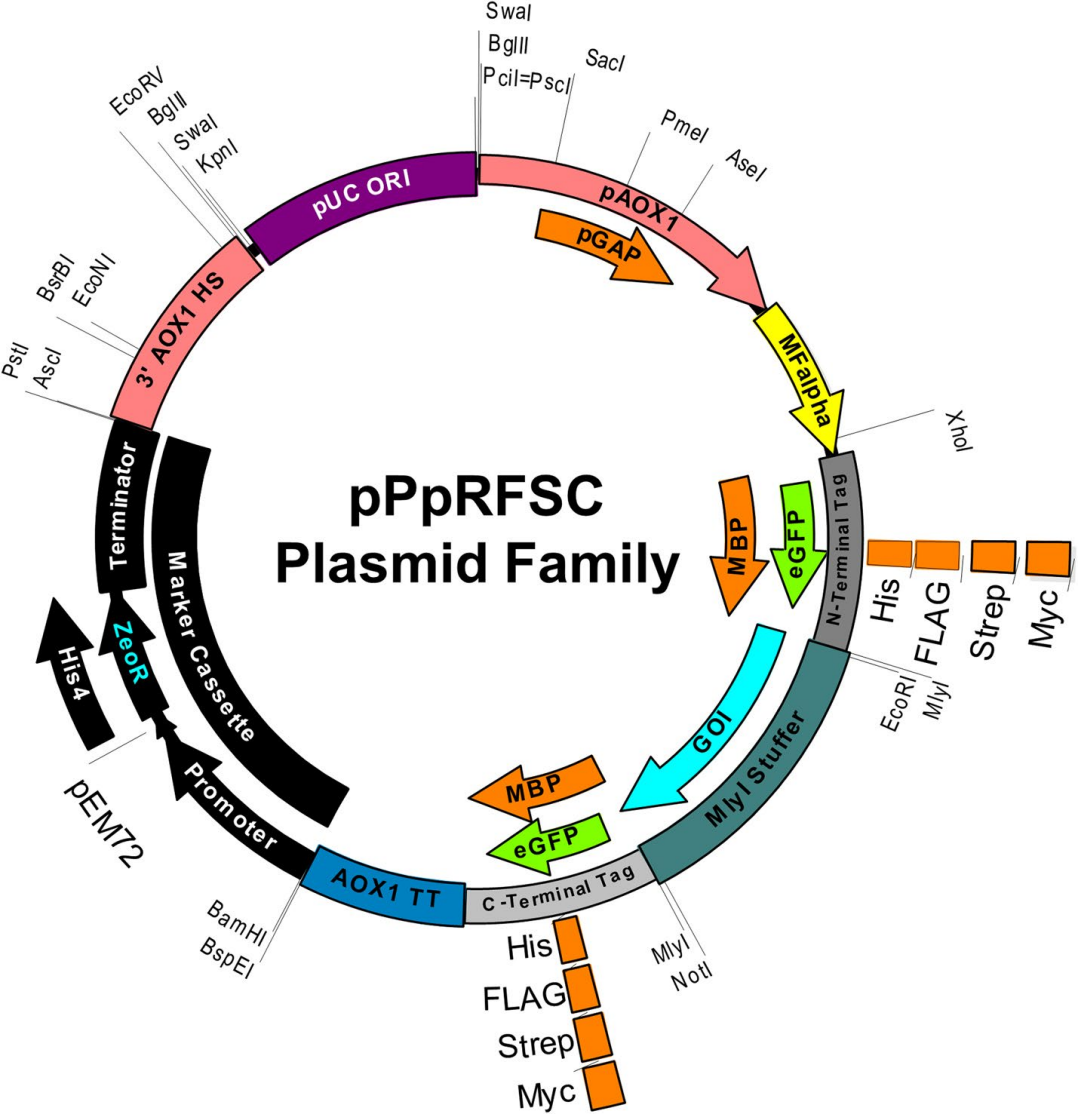
Representative map of *P. pastoris* RSFC plasmids. The features of all RSFC plasmids designed are summarized in this schematic map. Different promoters, N/C-terminal tags, resistance markers are shown. p*GAP* plasmids do not contain the 3′ *AOX1* homologous sequence for recombination (3′ AOX1 HS). HIS4 vectors contain in addition an ampicillin resistance cassette. The mating factor alpha signal sequence (MF alpha) is optional and only present in secretory plasmids. See Table 1 for the part combinations created in this study. Features are not drawn to scale. Exact plasmid maps are provided in the Additional file 3 in GenBank format.

We designed a set of 40 RSFC plasmids for *P. pastoris* (termed pPpRSFC) offering different tags (Myc, FLAG, His, Strep) and fusion proteins (enhanced green fluorescent protein, eGFP and maltose binding protein, MBP) in N- and C-terminal position, see Table 1 for exact properties and Figure 2 for a schematic vector map. We have assigned numbers (#1 to #40) to the plasmids and are using them hereafter when referring to a specific construct.

**Table 1:** RSFC vector family designed for *P. pastoris*

#	Name	Tag/Fusion protein, position^a^ and length^b^			TEV protease cleavage site	Mode of expression	EAEA repeat	Selection marker^c^	Plasmid size (bp)
1	PPpRSFC	–	–	–	NA	Intracellular	NA	Zeocin	4,840
2	pPpRSFC-MFalpha	–	–	–	NA	Secretory	Yes	Zeocin	5,104
3	pPpRSFC-MFalpha-noEAEA	–	–	–	NA	Secretory	No	Zeocin	5,092
4	pPpRSFC-pGAP	–	–	–	NA	Intracellular	NA	Zeocin	3,771
5	pPpRSFC-pGAP-MFalpha	–	–	–	NA	Secretory	Yes	Zeocin	4,035
6	pPpRSFC-pGAP-MFalpha-noEAEA	–	–	–	NA	Secretory	No	Zeocin	4,023
7	pPpRSFC-N-eGFP	eGFP	N	240	No	Intracellular	NA	Zeocin	5,584
8	pPpRSFC-C-eGFP	eGFP	C	239	No	Intracellular	NA	Zeocin	5,584
9	pPpRSFC-MFalpha-N-eGFP	eGFP	N	239	No	Secretory	Yes	Zeocin	5,848
10	pPpRSFC-MFalpha-C-eGFP	eGFP	C	239	No	Secretory	Yes	Zeocin	5,848
11	pPpRSFC-N-Myc	MYC	N	11	No	Intracellular	NA	Zeocin	4,870
12	pPpRSFC-C-Myc	MYC	C	10	No	Intracellular	NA	Zeocin	4,870
13	pPpRSFC-MFalpha-N-Myc	MYC	N	10	No	Secretory	Yes	Zeocin	5,134
14	pPpRSFC-MFalpha-C-Myc	MYC	C	10	No	Secretory	Yes	Zeocin	5,134
15	pPpRSFC-N-FLAG	FLAG	N	9	No	Intracellular	NA	Zeocin	4,864
16	pPpRSFC-C-FLAG	FLAG	C	8	No	Intracellular	NA	Zeocin	4,864
17	pPpRSFC-MFalpha-N-FLAG	FLAG	N	8	No	Secretory	Yes	Zeocin	5,128
18	pPpRSFC-MFalpha-C-FLAG	FLAG	C	8	No	Secretory	Yes	Zeocin	5,128
19	pPpRSFC-N-His-ncs	His	N	7	No	Intracellular	NA	Zeocin	4,858
20	pPpRSFC-C-His-ncs	His	C	6	No	Intracellular	NA	Zeocin	4,858
21	pPpRSFC-MFalpha-N-His-ncs	His	N	6	No	Secretory	Yes	Zeocin	5,122
22	pPpRSFC-MFalpha-C-His-ncs	His	C	6	No	Secretory	Yes	Zeocin	5,122
23	pPpRSFC-N-His	His	N	7	Yes	Intracellular	NA	Zeocin	4,879
24	pPpRSFC-C-His	His	C	6	Yes	Intracellular	NA	Zeocin	4,879
25	pPpRSFC-MFalpha-N-His	His	N	6	Yes	Secretory	Yes	Zeocin	5,143
26	pPpRSFC-MFalpha-C-His	His	C	6	Yes	Secretory	Yes	Zeocin	5,143
27	pPpRSFC-N-MBP	MBP	N	367	Yes	Intracellular	NA	Zeocin	5,959
28	pPpRSFC-C-MBP	MBP	C	366	Yes	Intracellular	NA	Zeocin	5,959
29	pPpRSFC-MFalpha-N-MBP	MBP	N	366	Yes	Secretory	Yes	Zeocin	6,223
30	pPpRSFC-MFalpha-C-MBP	MBP	C	366	Yes	Secretory	Yes	Zeocin	6,223
31	pPpRSFC-N-Strep	Strep	N	9	Yes	Intracellular	NA	Zeocin	4,885
32	pPpRSFC-C-Strep	Strep	C	8	Yes	Intracellular	NA	Zeocin	4,885
33	pPpRSFC-MFalpha-N-Strep	Strep	N	8	Yes	Secretory	Yes	Zeocin	5,149
34	pPpRSFC-MFalpha-C-Strep	Strep	C	8	Yes	Secretory	Yes	Zeocin	5,149
35	pPpRSFC-HIS	–	–	–	NA	Intracellular	NA	HIS4	7,683
36	pPpRSFC-HIS-MFalpha	–	–	–	NA	Secretory	Yes	HIS4	7,947
37	pPpRSFC-HIS-MFalpha-noEAEA	–	–	–	NA	Secretory	No	HIS4	7,935
38	pPpRSFC-HIS-pGAP	–	–	–	NA	Intracellular	NA	HIS4	6,614
39	pPpRSFC-HIS-pGAP-MFalpha	–	–	–	NA	Secretory	Yes	HIS4	6,878
40	pPpRSFC-HIS-pGAP-MFalpha-noEAEA	–	–	–	NA	Secretory	No	HIS4	6,866

*NA* not applicable, *ncs* no TEV protease cleavage site.

^a^N- or C- terminal fusion to the POI.

^b^Length in amino acids (intracellular N- terminal tags are because of the start codon one aa longer, the TEV protease cleavage site (seven aa) is not included in this number).

^c^Zeocin selection is applicable for *E. coli* and *P. pastoris*, HIS4 plasmids are based on ampicillin selection in *E. coli* and used in combination with a histidine auxotrophic (*his4*) *P. pastoris* strain.

After stuffer removal by *Mly*I digestion, a single PCR product can be cloned in a seamless and sequence independent fashion into all vectors, fused to tags and fusion proteins ranging from 18 to 1,101 bp in length. Epitope and affinity tags are included and constructs with affinity tags contain a TEV protease cleavage site to allow tag removal. The hexameric His tag is provided with and without TEV protease cleavage site. MBP is provided as a fusion protein with the potential to improve solubility and act as a purification tag, although in *P. pastoris* problems with proteolytic degradation have been reported [33]. eGFP is an enhanced version of GFP allowing simple fluorescence detection of tagged proteins.

When cloning a GOI into the pPpRSFC vectors, the forward primer must be designed starting at the DNA sequence coding for the 2nd amino acid of the POI (omitting the ATG start codon). The reverse primer must be designed starting (on the reverse strand) at the DNA sequence coding for the last amino acid/penultimate codon (omitting the stop codon). Especially a stop codon on the PCR product would interfere with tag fusions, therefore the start and stop codon are always provided on the vectors and must be omitted from PCR inserts.

*P. pastoris* is not only suitable for intracellular expression but can also produce secreted heterologous proteins at high titers while secreting only little endogenous protein [27–29]. Therefore we designed all plasmids also for secretory expression using the *S. cerevisiae* mating factor alpha pre-pro signal sequence (MF alpha), the most commonly applied signal sequence in *P. pastoris*. The MF alpha sequence is processed by two proteases (Ste13 and Kex2) that cleave the amino acid sequence KREAEA at the end of MF alpha [34]. Kex2 cleaves efficiently after KR whereas the Ste13 cleavage after the EA repeat may be incomplete, depending on the following amino acids of the POI. In several cases removal of the EAEA repeats has led to a more homogenous product [35, 36]. Therefore we designed the basic MF alpha pPpRSFC plasmids (#2, 3, 5, 6, 36, 37, 39, 40) with and without the EAEA sequence. Plasmids bearing tags always contain the EAEA repeat (Table 1).

#### Promoters, integration events and resistance markers

The pPpRSFC plasmids are based on the pPpT4 vector family reported by Näätsaari et al. [30] and also used as a platform for the *P. pastoris* Electra vectors by DNA2.0. The majority of pPpRSFC plasmids contain the promoter of the *alcohol oxidase 1* gene (p*AOX1*). This strong, tightly regulated methanol inducible promoter is most commonly used in *P. pastoris* [37]. We have also designed basic plasmids bearing the *glyceraldehyde*-*3*-*phosphate dehydrogenase* promoter (p*GAP*) to enable methanol free, constitutive expression (see Table 1).

In contrast to the yeast *S. cerevisiae*, where stable, autonomously replicating plasmids are available, circular plasmids bearing a yeast ARS (autonomously replicating sequence) are not stable in *P. pastoris* and genomic integration of plasmid cassettes is the method of choice for heterologous gene expression [27, 38]. Most commonly *P. pastoris* integration cassettes are created by linearizing plasmids or generation of linear cassettes by PCR [39, 40] and targeted to the *AOX1* locus via homologous sequences. Depending on the linearization site in the plasmid, different homologous recombination events can be targeted [38]. The pPpRSFC plasmids allow linearization to target gene replacement at the *AOX1* locus. Thereby the endogenous *AOX1* gene is deleted and the minor *AOX2* gene must take over the function of oxidizing methanol to formaldehyde. Due to the lower expression levels of *AOX2*, these *aox1* knockout strains show a Mut^S^ (methanol utilization slow) phenotype, which may result in higher yields than a Mut^+^ phenotype [41, 42]. This can be achieved by linearization using *Bgl*II. If the *Bgl*II site is present in the insert, the vectors can still be linearized using the rare 8 bp *Swa*I sites as a failsafe backup. If a Mut^+^ phenotype is desired, the vectors can be linearized using unique REs cleaving in the 5′ or 3′ homologous sequence (e.g. *Pme*I, *Ase*I or *Eco*NI, *Bsr*BI). However, due to low homologous recombination frequencies in *P. pastoris* wildtype strains [30], even when targeting a gene replacement at the *AOX1* locus (expected Mut^S^ phenotype), still the majority of transformants are Mut^+^. Therefore it is necessary to validate the Mut phenotype by growth on methanol plates.

The RSFC plasmids are based on a modular design, the promoter, N- or C-terminal tags, terminator, the resistance marker and the 3′ homologous sequence can be easily exchanged using unique restriction enzyme sites (Figure 2). Most plasmids are based on Zeocin selection, however basic expression plasmids (#35–40) were also constructed with a histidine marker to be used with auxotrophic strains. The tagged expression cassettes from the Zeocin plasmids can be easily shuffled to the histidine plasmid backbones using unique *Pci*I and *Bam*HI sites.

In the pPpRSFC plamids not only the transition between the insert and the vector is seamless, also the transition of the promoter to the start codon and the stop codon to the terminator are seamless. In standard RE based cloning, the MCS may interfere with translation initiation [43] and this appears relevant for the *AOX1* promoter as extensions of the 5′ UTR (also caused by a MCS) were shown to negatively affect expression [44].

### Applications of RSFC vectors to optimize HRP expression in *P. pastoris*

#### Effects of tags and fusions proteins in N- and C-terminal position

**Figure 3:**
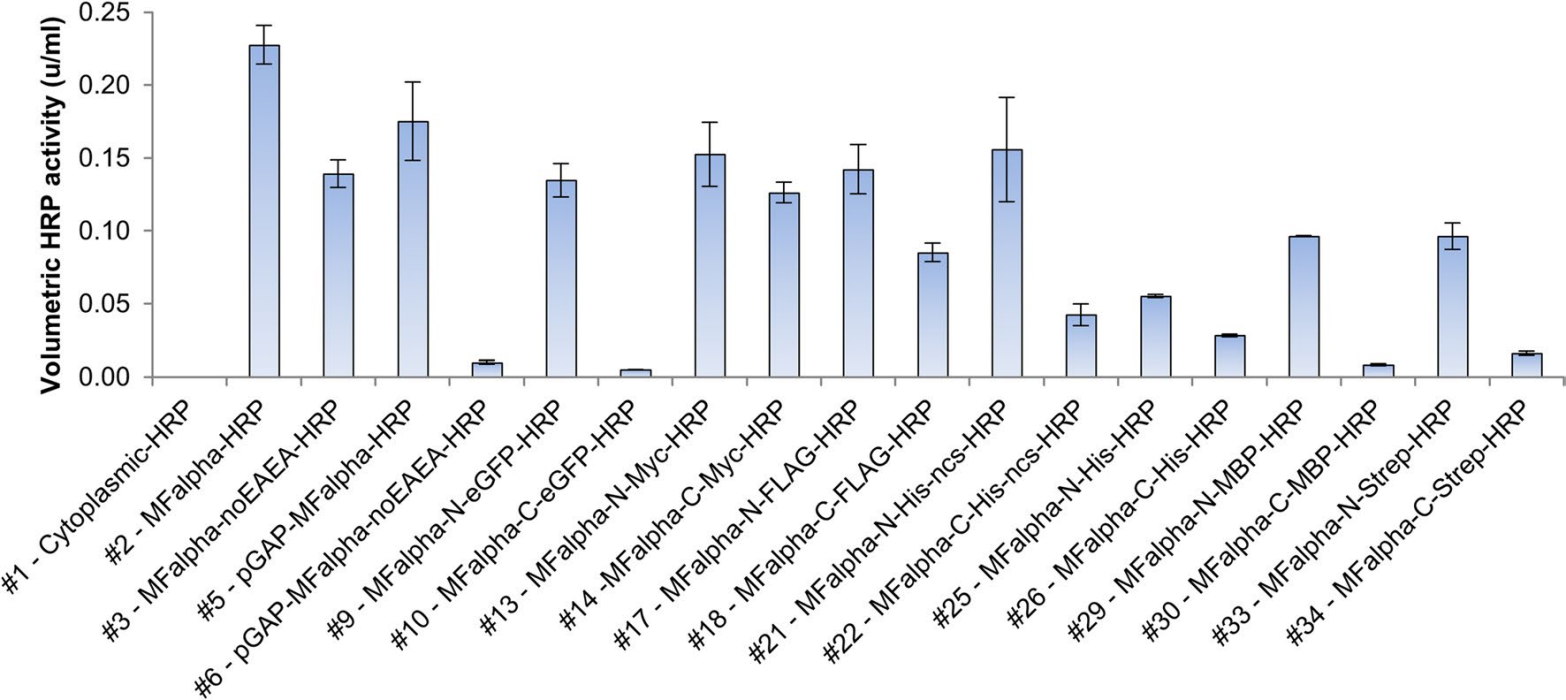
Type of tag and position (N/C-terminal) strongly affect volumetric HRP activities. Volumetric HRP activities in the supernatant with ABTS as substrate after cultivation on methanol for 72 h are shown. The pPpRSFC plasmids used are indicated via the *numbers* given in Table 1, the use of a signal sequence (MFalpha), different promoter than p*AOX1* and if applicable tag and position (N/C) are given. Mean values and standard deviations of biological triplicates are shown.

With the set of pPpRSFC plasmids available, we aimed to validate the system with a typical application. We tested expression of horseradish peroxidase (HRP), a commonly used reporter enzyme for signal amplification in diagnostic kits and immunohistochemistry. Secretory expression of HRP has been previously demonstrated in *P. pastoris* [42, 45–47]. Cytoplasmic expression promised little chance of success as HRP is a secretory plant peroxidase that requires formation of disulfide bridges and is typically glycosylated in the secretory pathway [48, 49]. Still, we tested the basic pPpRSFC plasmid (#1, untagged, p*AOX1*) for cytoplasmic HRP expression. This construct showed neither activity in the supernatant (Figure 3) nor in the cytoplasm (data not shown). Therefore different tags were only evaluated for secretory expression. A single PCR fragment of HRP was cloned into the vectors as outlined above. All p*AOX1* plasmids were linearized via *Bgl*II sites to target a gene replacement event at the *AOX1* locus, and screened for a Mut^S^ phenotype, which has been reported to be more favorable for HRP expression than Mut^+^ [42].

The different tags and positions had diverse effects on volumetric HRP activities (Figure 3) and led to valuable insights. For all tags, the N-terminal version was giving higher activities than the C-terminal version. For the larger fusion proteins (eGFP and MBP), C-terminal tagging even led to almost complete loss of activity (#10 and #30). Comparing the tagged construct with the highest activity (#21) with the construct of the lowest activity (#10) gives a 31 fold difference. N-terminal tagging with the relatively large eGFP (and MBP) did not strongly affect activity, whereas shorter tags (Myc, FLAG, His, Strep) showed varying effects. The N-terminally His tagged construct with TEV protease cleavage site (#25) showed the lowest activity of all N-terminally tagged proteins. However, the N-terminal His tagged construct without TEV protease cleavage site (#21) showed activity similar to other tags, hinting a negative effect of the TEV protease cleavage site in this context. Changes of the MFalpha sequence by removal of the EAEA sequence decreased activity 1.6 fold with the methanol inducible *AOX1* promoter (#2 vs. #3). With the constitutive *GAP* promoter (#5 vs. #6), removal of the EAEA sequence even led to a 17 fold decrease in activity. A possible mechanistic explanation would be that the EAEA repeats improved secretion due to increased Kex2 cleavage efficiencies [50, 51]. p*GAP* driven HRP expression was therefore, depending on the presence of EAEA repeats, competitive to the methanol inducible p*AOX1*. Due to shorter process times (not requiring methanol induction) p*GAP* driven expression may even be more favorable for HRP production regarding space time yields and process setup.

The effects seen on volumetric activities by using different tags may be caused by various reasons. On the one hand the tags may have interfered to a different extent with protein folding or access of the substrate to the active site thereby negatively affecting activity. On the other hand they also may have affected the protein yields by altering the protein stability, interfering with the secretion process or even on the mRNA level with transcript stability. Also the tags or linker sequence may have targeted proteolytic degradation, as previously reported for MBP in *P. pastoris* [33]. However, as we aimed only to evaluate the suitability of the RSFC strategy for screening different tags, we did not further investigate the underlying causes. The pPpRSFC plasmid family proved to be a simple tool to optimize volumetric activities of tagged HRP, showing that especially the tag positions and presence of EAEA repeats are crucial factors.

#### Fluorescence microscopy of strains expressing eGFP tagged HRP

GFP has routinely been used in *P. pastoris* as an intracellular reporter for comparing promoter activities [52–54] and to facilitate screening of protein production by testing fusions proteins [55], especially for membrane proteins [56–58]. Concerning GFP fusions of secretory proteins, conflicting results were obtained. In some cases GFP was successfully used as secretion reporter and for protein fusions [59–62]. In other cases problems with secretion (e.g. intracellular retention) were noticed [63–66]. As we had also designed N- and C-terminal fusions with eGFP (including the MFalpha signal sequence for secretion, #9 and #10), we performed fluorescence microscopy to investigate possible cellular retention and bottlenecks in the HRP secretion process.

**Figure 4:**
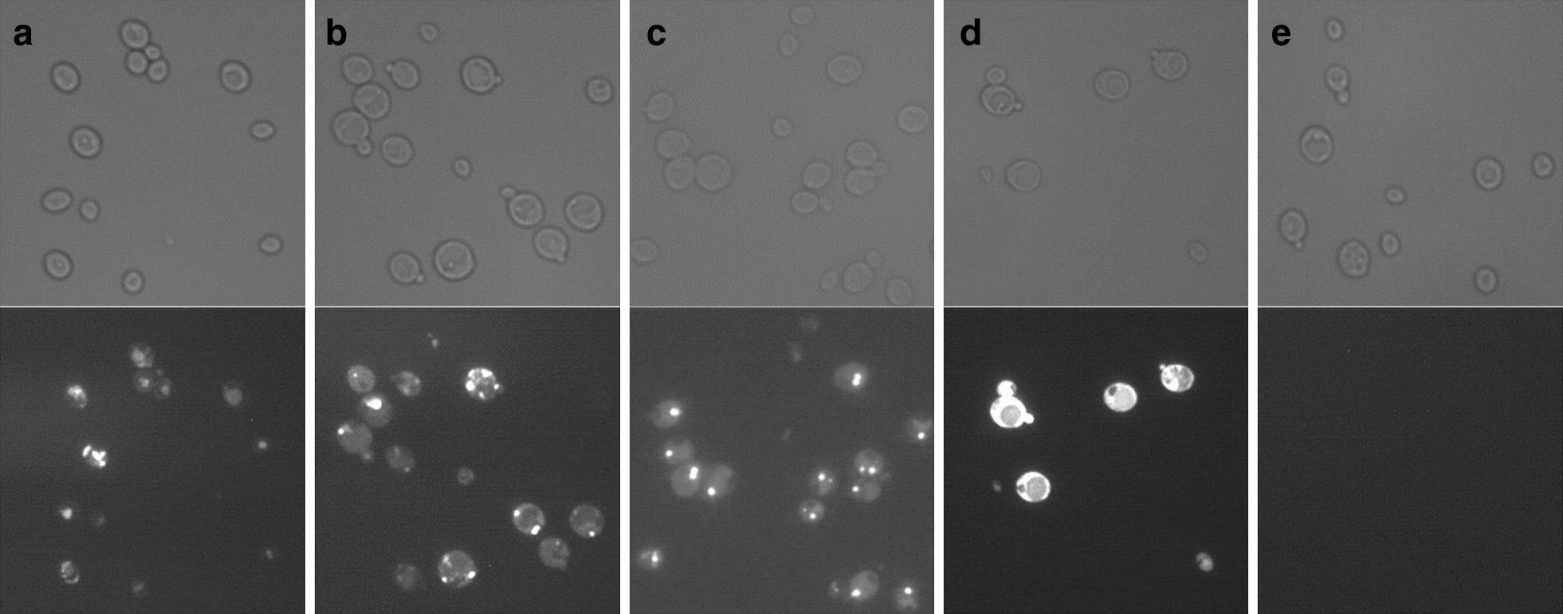
Fluorescence microscopy of fusions of HRP to GFP. Bright field images are shown on *top*, fluorescence images *below.* **a** HRP N-terminally tagged with eGFP (#9-MFalpha-N-eGFP-HRP), **b** HRP C-terminally tagged with eGFP (#10-MFalpha-C-eGFP-HRP), **c** control of eGFP with MFalpha (self-ligated #9), **d** control of cytoplasmic eGFP expression (#1-eGFP), **e** negative control of empty Mut^S^ strain. For the bright field image of panel (**c**) brightness was decreased −11%, contrast was increased +44% for better comparability with the other panels.

The N-terminal eGFP-HRP fusion exhibited largely unchanged HRP activity, whereas the C-terminally tagged version had almost completely lost activity (Figure 3). We also included controls of intracellular eGFP expression (#1) and secretory eGFP alone (without an HRP fusion, created by self-ligating #9). Fluorescence microscopy images of methanol grown cells are shown in (Figure 4). While cytoplasmic expression showed bright fluorescence of the whole cell (Figure 4d), all secretory constructs (Figure 4a–c) showed punctate structures. These structures appeared somewhat similar to ER or Golgi mistargeting observed previously when expressing a GFP tagged membrane protein (human µ-opioid receptor, a G-protein coupled receptor) [65]. Most notably also the control of eGFP alone (Figure 4c), without an HRP fusion showed this retention. eGFP was apparently poorly secreted in *P. pastoris* and effects evoked by HRP may be masked and outweighed by the poor eGFP secretion.

We also measured eGFP fluorescence in the supernatant and the cell fraction (Additional file 4: Figure S3). Fluorescence in the supernatant could be detected for secretory constructs (Additional file 4: Figure S3a–c), while the cytoplasmic eGFP expression control (Additional file 4: Figure S3d) showed only marginal fluorescence in the supernatant. However, also for the secretory constructs (Additional file 4: Figure S3a–c) intracellular fluorescence surpassed fluorescence in the supernatant approximately 5- to 12-fold. These results suggested together with the microscopy images, that large amounts of eGFP were withheld in the secretion process. In this respect, eGFP fusion proteins may be used with caution when investigating secretory processes in *P. pastoris*. However, these effects may also be protein dependent, as there were cases reported where GFP was successfully used to evaluate signal sequences [59, 60] and some GFP fusion proteins were sufficiently secreted [61, 62].

## Conclusions and outlook

The RSFC cloning strategy outlined here and the pPpRSFC plasmid family are simple tools to optimize expression of tagged proteins with little cloning efforts. RSFC requires at first the design and assembly of the vector family to be used. However, subsequent screening is drastically facilitated as large amounts of vector backbones can be prepared at once by *Mly*I digestion. Subsequently, the backbones ready for cloning can be frozen as aliquots and thawed when needed.

There have been systems reported previously that allow testing of the expression of a POI in different expression hosts by using only two PCR products [67]. This approach is based on ligation-independent cloning (LIC) similar to [10–13, 68]. While these methods allow highly efficient, seamless cloning, they rely on the annealing of single stranded overhangs, thereby requiring identical sequences between vector and insert. Therefore these methods are not suitable for seamless, sequence independent fusions possible with RSFC. However, as a downside of RSFC the blunt end ligations work less efficiently than annealing based in vitro recombination methods [10–13] and confirmation of the correct orientation is required. Otherwise only about 50% of the transformants show the desired orientation which is a disadvantage for library approaches. Nevertheless, after stuffer removal, inserts can also be cloned directionally into RSFC plasmids by in vitro recombination methods (such as Gibson assembly [12]). However this task requires the design of a separate primer for every tag and position to be tested as the overhang required for annealing changes between the vectors. We recommend using in vitro recombination methods with pPpRSFC plasmids when testing only a few constructs. When testing a larger number of constructs, the increased costs for primers and materials associated with in vitro recombination methods outweigh the costs for cPCRs to test the orientation of blunt end ligations. When performing a single experiment and cloning a low number of GOIs with only one tag, it will be more effort to set up a RSFC vector than to order a few long primers. However, if routinely a large number of GOIs should be screened with a set of tags in different positions, RSFC vectors are a powerful strategy.

A limitation of the RSFC system reported here is the use of *Mly*I, the only type IIS enzyme performing a blunt end cleavage. The recognition sequence of *Mly*I is five bp long (Figure 1a), thereby posing a problem as it appears statistically once per 512 bp (4^5^/2) [69]. This may require frequent removal of *MlyI* sites in the vector backbones to be used. *Mly*I sites in CDSs of tags, fusion proteins and resistance markers can be easily removed by mutating the *Mly*I sequence to synonymous codons. However, mutating sites present in promoters, terminators or origins of replication have to be validated for unchanged functionality (or must be exchanged for parts free of *Mly*I sites). These issues could be solved by using artificial type IIS REs with longer recognition sequences. The catalytic domain of the archetypical type IIS enzyme *Fok*I has been fused to I-*Sce*I, a homing endonuclease with an 18 bp recognition sequence. This chimeric meganuclease showed sufficient cleavage resulting in 4 bp ‘sticky’ overhangs that could be ligated at 90% fidelity [69]. Following this strategy, the catalytic domain of *Mly*I (which is similar to *Fok*I [70]) could also be fused to I-*Sce*I. Statistically an 18 bp recognition sequence would appear approximately once in 10^11^ (4^18^) bp, however I-*Sce*I recognizes also slightly degenerate sequences leading to an estimated appearance once in 10^8^ bp [69, 71, 72], which would still surpass the specificity of wildtype *Mly*I by several orders of magnitude.

Most vectors for *P. pastoris* have been conceptualized solely as straightforward expression vectors ([8, 22, 25, 30, 31] and *P. pastoris* plasmids by Life Technologies, BioGrammatics and DNA2.0) and few plasmid families allow to fine-tune expression [30, 31]. The 40 plasmids reported here extend the scope of applications and facilitate characterization and optimization of the production of heterologous proteins in *P. pastoris*. The RSFC strategy outlined here is not limited to tags and fusions proteins, but could also be applied to compare different promoters or signal sequences in other expression systems. Similarly, isoenzymes or families of homologous enzymes can be fused to tags to screen for better expression, solubility or other properties to identify enzymes combining desired biological, chemical and technological features.

## Materials and methods

### Chemicals and media

Phusion DNA Polymerase, restriction endonucleases and other DNA modifying enzymes were acquired from Thermo Fisher Scientific (Waltham, MA, USA) or New England Biolabs (Ipswich, MA, USA). Miscellaneous chemicals were purchased from Becton, Dickinson and Company (Franklin Lakes, NJ, USA), Carl Roth (Karlsruhe, Germany) and Fresenius Kabi Austria (Graz, Austria).

Plasmids were isolated using a GeneJET Plasmid Miniprep Kit by Thermo Fisher Scientific. The standard protocol was optimized for *Mly*I based constructs to compensate the decreased plasmid yields. A single colony of a strain bearing the respective plasmid was streaked on an agar plate containing the respective antibiotic. After incubation overnight, a cell pellet (approximately 0.1 g wet cells) was scratched of the plate and used for the isolations (final elution volume: 100 µl of ddH_2_O).

Agarose embedded DNA, restriction digests and PCRs were purified using a Wizard SV Gel and PCR Clean-Up System by Promega.

*P. pastoris* strains were grown on full medium (yeast extract, peptone, 2% glucose, YPD), buffered minimal dextrose (BMD) and buffered minimal methanol medium with 0.5% methanol (BMM) as described by Weis et al. [16]. As only exception we used 2% glucose in the BMD medium and for HRP expression, media were supplemented with 1 mmol/l ferrous sulfate heptahydrate (FeSO_4_.7H_2_O). *Escherichia coli* strains were selected on LB-medium containing 25 μg/ml Zeocin™ (Life Technologies, Carlsbad, CA, USA). *P. pastoris* transformants were selected on YPD agar plates containing 100 μg/ml Zeocin. Primers were ordered from Integrated DNA Technologies (Leuven, Belgium), see Additional file 5: Table S1 for the sequences.

### Plasmid construction

#### *pombe* RSFC test vectors pGAZ2-TA-BmrI-stuffer and pGAZ2-Blunt-MlyI-stuffer

For all cloning work an *E. coli* Top10 F’ strain was used. The vectors for initially comparing blunt end and TA cloning were based on a replicative *S. pombe* vector pGAZ2 (Additional file 1: Figure S1, unpublished results). For the TA-cloning vector ‘pGAZ2-TA-BmrI-stuffer‘, a stuffer fragment was amplified using primers TA_fwd_HindIII+BmrI+stuffer and TA_rev_BamHI+BmrI+stuffer (see Additional file 5: Table S1) and cloned into pGAZ2 via *Hind*III and *Bam*HI sites. The stuffer fragment was selected as a sequence that has no significant homology to *E. coli* and *S. pombe* genomes and lacks *Mly*I, *Bmr*I, *Hind*III and *Bam*HI RE sites; we used a part of a *P. pastoris* alpha, alpha trehalase gene. The ‘pGAZ2-Blunt-MlyI-stuffer’ vector required mutating two *Mly*I sites in the vector backbone. This was done by PCR amplifying the vector using primers pUC_mut_MlyI_fwd + pUC_mut_MlyI_rev and ZeoCDS_mut_MlyI_fwd + ZeoCDS_mut_MlyI_rev using Pfu Ultra polymerase (Agilent Technologies, Santa Clara, CA) followed by *Dpn*I digestion to remove template vector. The *Mly*I site in the pUC was mutated to the sequence reported by Rao et al. [25], the *Mly*I site in the zeocin resistance gene was mutated to a synonymous codon. After transformation, introduction of the correct mutations were confirmed by Sanger sequencing. Both plasmids do not provide seamless fusions, as the stuffer fragments were for convenience inserted via *Hind*III and *Bam*HI sites leaving RE site scars. For test purposes the gene coding for *Thermomyces lanuginosus* endo-beta-1,4-D-xylanase was amplified using primers Xyla_fwd and Xyla_rev and cloned into the two vectors (detailed protocol see below).

#### P. pastoris pPpRSFC plasmid family

The expression plasmids used in this study are based on the pPpT4 *P. pastoris*/*E. coli* shuttle vector family (e.g. GenBank accession number JQ519690.1) reported by Näätsaari et al. [30]. Two *Mly*I sites in the backbone (pUC and zeocin resticane gene) were mutated in the same way as in the *S. pombe* plasmids of this study (Additional file 1: Figure S1; same primers as in Additional file 5: Table S1) and confirmed by sequencing. The *AOX1* promoter, *Mly*I stuffer fragment and *AOX1* terminator were amplified in separate PCR reactions using primers PAOX1_PciIF/OePAox1StufferR, OestufferF/OeStufferR and OeAox1TTstufferF/Aox1TT_BamHIR respectively. In the subsequent overlap extension PCR reactions the fragments were joined together using primer pair PAOX1_PciIF/AOX1TT_BamHIR followed by restriction with *Pci*I/*Bam*HI and were cloned in a vector backbone with mutated *Mly*I sites to create an intermediatory plasmid backbone termed ‘pPp’. The stuffer fragment sequence was selected from as a sequence that has no homology to *E. coli* and *P. pastoris* and lacks unique RE used in the pPpRSFC plasmid family. We selected a part of a gene involved in the *S. cerevisiae* biotin metabolism. An *Eco*RI site in the stuffer was mutated using primers pairs OeEcoRIF and OeEcoRIR. There appeared a few additional mutations in the stuffer that had no functional consequences and where therefore left unchanged (see plasmid sequences in Additional file 3).

For constitutive plasmids, the *GAP* promoter was amplified via primers GAP_PciIF/OeGapStuffR and was cloned into the pPp backbone using PciI/EcoRI to create #4 (pPpRSFC-pGAP). The 3′ AOX1 homologous sequence was amplified via primers 3′AOX1_PstIASCIF/3′AOX1_KpnISwaIR and was cloned into pPp using *Kpn*I/*Pst*I restriction sites to create #1 (pPpRSFC). For secretory expression plasmids, the MFAlpha sequence was amplified using primer pair AlphaFSSF/AlphaEcoRIR (or aEAEAEcoRIR for insertion of Glu-Ala repeats). The *AOX1*/*GAP* promoters were amplified via primers PAOX1_PciIF + OeAlphaPAox1R/GAP_PciIF+ OeGapAlphaR. The MFAlpha sequence was fused with p*AOX1*/p*GAP* using primers PAOX1_PciIF+ AlphaEcoRIR (expression cassette for #3) or PAOX1_PciIF+ aEAEAEcoRIR (expression cassette for #2),/GAP_PciIF+ AlphaEcoRIR (expression cassette for #6) or GAP_PciIF+ aEAEAEcoRIR (expression cassette for #5). The pAOX1-MFAlpha PCR products were cloned into pPpRFSC via PciI/EcoRI sites to create #3 and #2. The pGap-MFAlpha fusion construct was cloned into pPpRSFC-pGAP via PciI/EcoRI restriction site to construct pPpRSFC-#6 and #5.

p*GAP* plasmids do not contain the 3′ *AOX1* sequence for homologous integration in the *AOX1* locus. Plasmids #1 to #6 were made initially and completely sequenced. In the creation of the following plasmids, only newly inserted parts (and the RE sites used for cloning) were confirmed by sequencing. A full description of how the 28 plasmids (#7–#40) with the N- and C- terminal tags were created would be beyond the scope of this section and is provided in the Additional file 5: Table S1 (spreadsheets on plasmid construction). For further details consult the annotated plasmid sequences provided in Additional file 3.

The HRP gene (isoenzyme A2A [46, 47]) was amplified using primers HRP-A2-RSFC-fwd and HRP-A2-RSFC-rev and cloned in the respective vectors (detailed protocol see below).

#### RSFC cloning of inserts and colony PCRs

For blunt end cloning, the vector backbone was dephosphorylated using either Thermo Scientific shrimp alkaline phosphatase or FastAP according to the manufacturer’s recommendations. The backbone was gel purified and used for ligations with phosphorylated PCR products. Prior, PCR primers were phosphorylated using Thermo Scientific T4 Polynucleotide Kinase according to the manufacturer’s recommendations. Subsequently the reaction mixtures containing the phosphorylated primers were desalted on nitrocellulose filters (Merck Millipore, Darmstadt, Germany) and added to the PCR (Phusion polymerase). Ligations were performed using the blunt end protocol provided for Thermo Scientific T4 DNA Ligase.

For TA cloning, phosphorylated Phusion PCR products were purified (Promega Wizard SV Gel and PCR Clean-Up System) and dA-tailed using Taq-Polymerase (GoTaq Flexi, Promega [Fitchburg, WI, USA], standard buffer, 0.2 mmol/l dATP, 30 min incubation at 72°C) and directly used for ligation (blunt end protocol provided for Thermo Scientific T4 DNA Ligase).

To verify the correct orientation by colony PCR, primers were selected as outlined in Additional file 2: Figure S2. A tiny amount of an *E. coli* colony from a transformation of the respective ligation was added to a GoTaq Flexi reaction. The manufacturer’s protocol was followed except reducing the reaction volume to 20 µl and increasing the initial denaturing step to five min to break the cells.

#### *P. pastoris* transformations and screening

For testing the pPpRSFC plasmids, the *P. pastoris* CBS7435 wildtype strain was used. Plasmids bearing the *AOX1* promoter were linearized with *Bgl*II, plasmids with p*GAP* were linearized with *Swa*I. All linearized plasmids were transformed into competent *P. pastoris* cells prepared by the condensed protocol reported by Lin-Cereghino et al. [73]. Only low amounts of DNA (0.5 µg) were used for the transformations to avoid multicopy integration. A landscape of 80 clones was screened and checked for the desired Mut^S^ phenotype on minimal methanol plates. Ten Mut^S^ clones were rescreened for uniform expression; a single representative clone was used for the subsequent characterizations. Screenings, rescreening and characterizations were performed in deep well plates as described previously [74]. BMD 2% was used instead of BMD 1% (giving higher yields, data not shown) and the methanol induction was performed in 12 h intervals for 72 h.

#### HRP activity assay, eGFP fluorescence microscopy and measurements

HRP activity assays with 2,2′-azino-bis(3-ethylbenzthiazoline-6-sulphonic acid) diammonium salt (ABTS) were performed as described previously [42]. For intracellular HRP activity measurements, cells were broken using Yeast Protein Extraction Reagent (Y-PER from Thermo Scientific).

The cell suspensions of eGFP expressing strains were centrifuged and washed in an equal amount of water before fluorescence microscopy (Leica Microsystems, Germany, DM LB2, DFC350FX) at 1,000-fold magnification, fluorescence images were taken using filter set ‘I3’ [excitation filter BP 450–490]. eGFP fluorescence (ex/em 488/507 nm) and OD_600_ were measured in a Synergy MX plate reader (Biotek, Winooski, VT, USA) using micro titer plates (Nunc MicroWell 96-Well Optical-Bottom Plates with Polymer Base, Black; Thermo Fisher Scientific). Cell suspensions were diluted to be within the linear range. The background measurements of diluted medium were subtracted. Subsequently the relative fluorescence units were normalized per OD_600_.

## Additional files

Additional file 1:
**Figure S1.** Vector maps of the *S. pombe* vectors used in this study Additional file 2:
**Figure S2.** Simple strategy for confirming the orientation of the insert. The forward or reverse primer used for amplifying the insert can be used together with the forward or reverse sequencing primer of the vector to confirm the correct orientation. Upon correct primer choice only the forward orientation gives a PCR fragment. The sequencing primers designed for Sanger sequencing allow sequencing of the insert from both sides. Depending on the vector, different primers should be used (e.g. when the MFalpha signal sequence or a fusion protein is present, see the primer list for all sequencing primers available). Additional file 3: Plasmid sequences of the constructs used in this study in Genbank format. Additional file 4:
**Figure S3.** Fluorescence measurements of fusions of HRP to eGFP. Samples are labeled in the same way as in Figure 4. eGFP fluorescence of supernatants and cell pellets of methanol induced cells were normalized per cell density (OD_600_). Additional file 5:
**Table S1.** Primers used in this study and detailed plasmid construction. Primers used for creating the *S. pombe* and *P. pastoris* plasmids are separated. Also primers for construction of the plasmids are separated from primers for sequencing and insertion of GOIs. In addition separate spreadsheets are providing information on the exact construction of the plasmids by listing the PCR products and restriction enzymes used for assembly.
