# Supplementary figures and images for: Restriction site free cloning (RSFC) plasmid family for seamless, sequence independent cloning in Pichia pastoris
Source: Microb Cell Fact. 2015 Jul 14;14:103. doi: 10.1186/s12934-015-0293-6 (PMC4501187; doi:10.1186/s12934-015-0293-6)

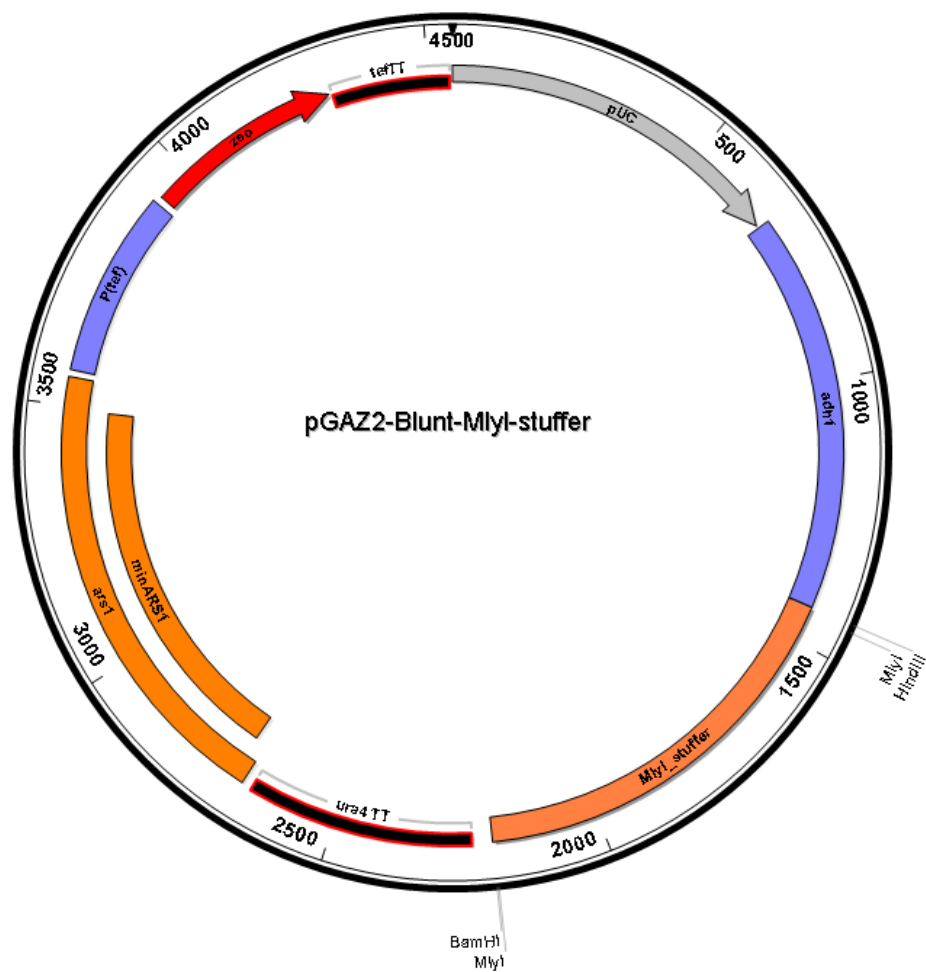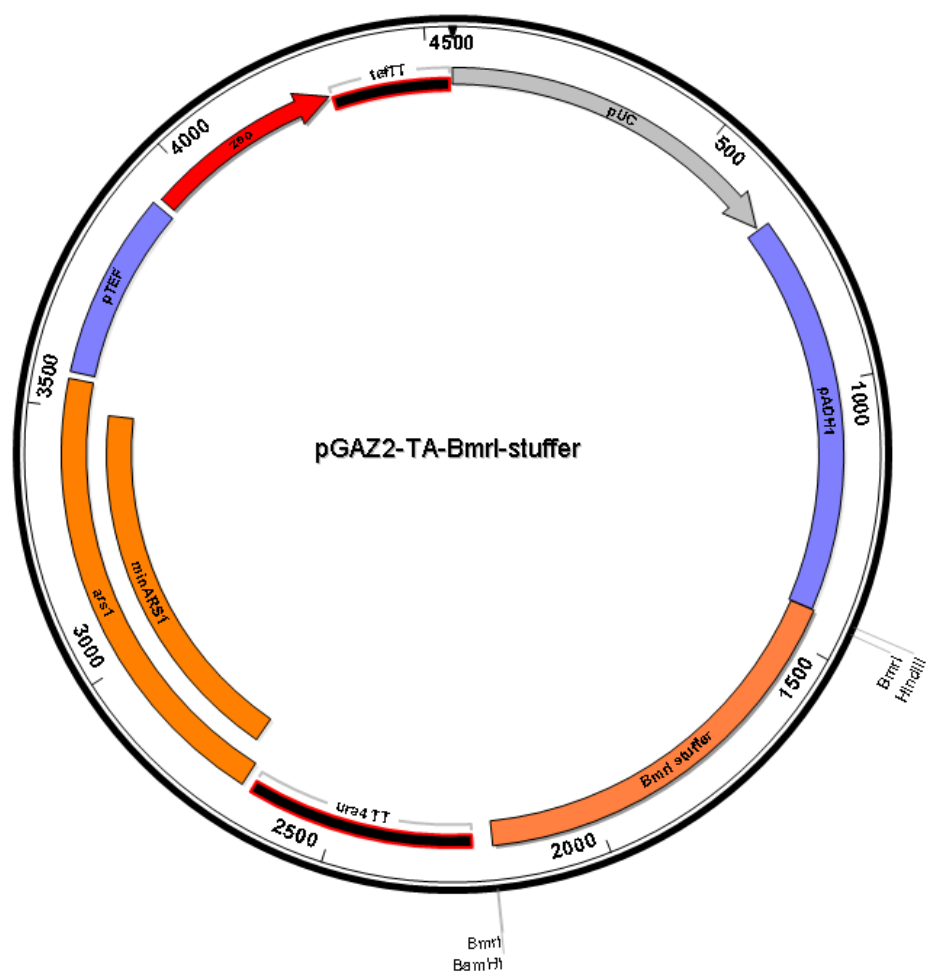

Supplement: Additional file 1: — Figure S1. Vector maps of the S. pombe vectors used in this study [file 12934_2015_293_MOESM1_ESM.pdf]

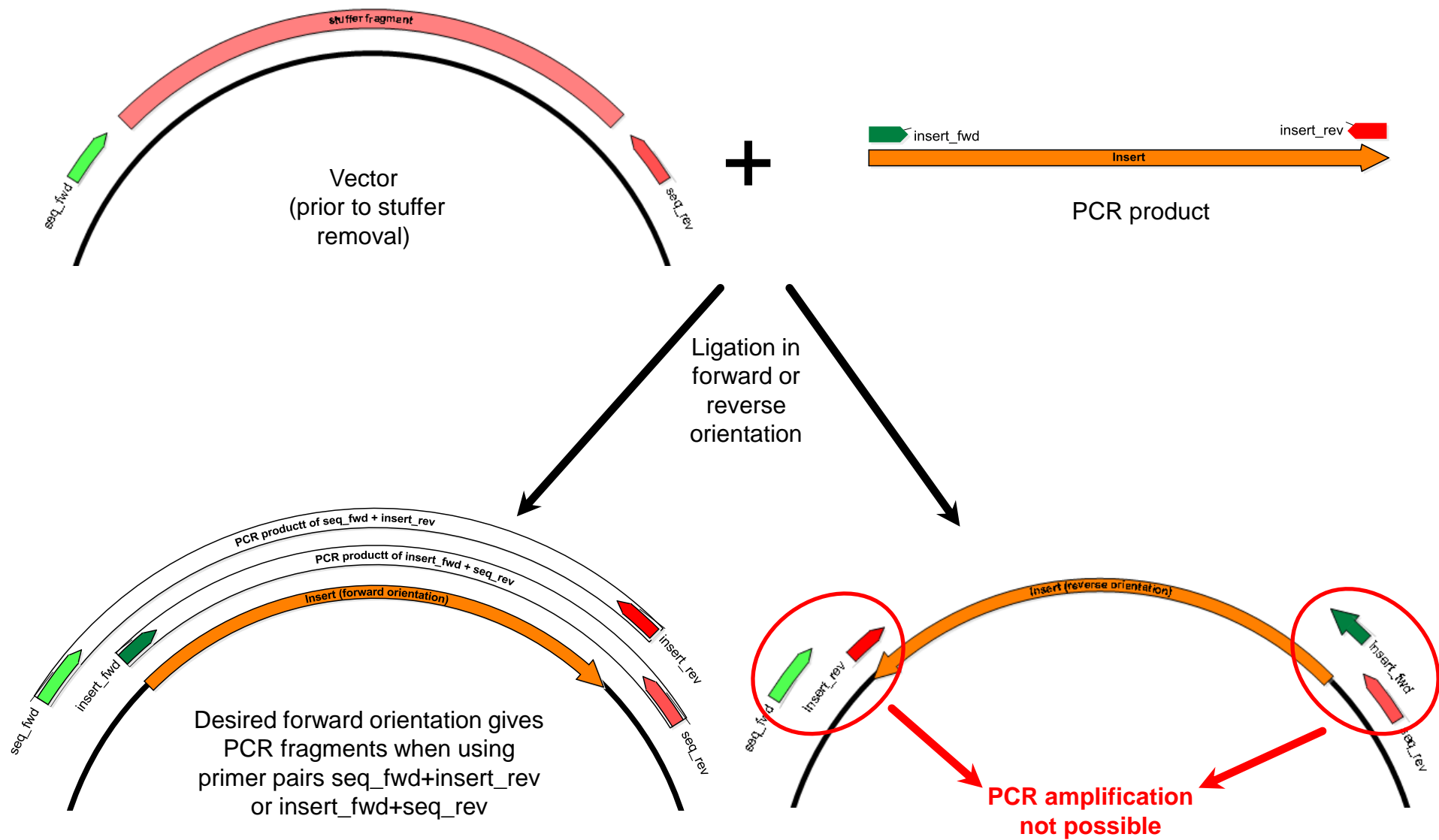

Supplement: Additional file 2: — Figure S2. Simple strategy for confirming the orientation of the insert. The forward or reverse primer used for amplifying the insert can be used together with the forward or reverse sequencing primer of the vector to confirm the correct orientation. Upon correct primer choice only the forward orientation gives a PCR fragment. The sequencing primers designed for Sanger sequencing allow sequencing of the insert from both sides. Depending on the vector, different primers should be used (e.g. when the MFalpha signal sequence or a fusion protein is present, see the primer list for all sequencing primers available). [file 12934_2015_293_MOESM2_ESM.pdf]

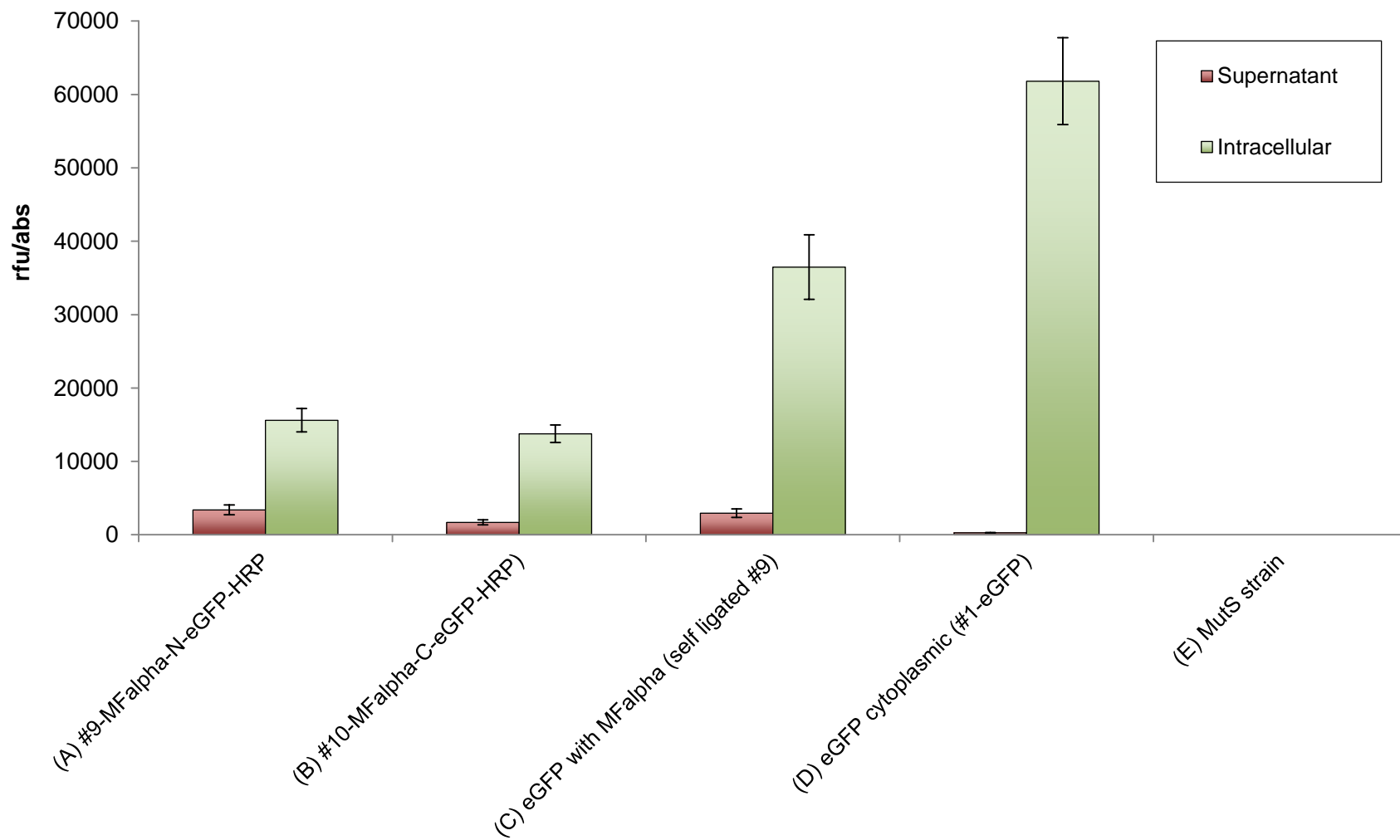

Supplement: Additional file 4: — Figure S3. Fluorescence measurements of fusions of HRP to eGFP. Samples are labeled in the same way as in Figure 4. eGFP fluorescence of supernatants and cell pellets of methanol induced cells were normalized per cell density (OD600). [file 12934_2015_293_MOESM4_ESM.pdf]
